# Salidroside attenuates HALI via IL-17A-mediated ferroptosis of alveolar epithelial cells by regulating Act1-TRAF6-p38 MAPK pathway

**DOI:** 10.1186/s12964-022-00994-1

**Published:** 2022-11-21

**Authors:** Baoyue Guo, Zhongfu Zuo, Xingwei Di, Ying Huang, Gu Gong, Bo Xu, Lulu Wang, Xiaoyu Zhang, Zhuang Liang, Yang Hou, Xuezheng Liu, Zhansheng Hu

**Affiliations:** 1 The Intensive Care Unit, The First Affiliated Hospital, Jinzhou Medical University, No. 2, Section 5, Renmin Street, Jinzhou, 121012 People’s Republic of China; 2 Liaoning Key Laboratory of Diabetic Cognitive and Perceptive Dysfunction, Department of Anatomy, Histology and Embryology, Jinzhou Medical University, Jinzhou, 121001 People’s Republic of China; 3 The Intensive Care Unit, The Central Hospital of Jinzhou, Jinzhou, 121001 People’s Republic of China; 4 The Intensive Care Unit, The Central Hospital of Dandong, Dandong, 118002 People’s Republic of China; 5 College of Biological Information Engineering, Jinzhou Medical University, Jinzhou, 121001 People’s Republic of China

**Keywords:** IL-17A, Ferroptosis, Salidroside, Immune cells, HALI

## Abstract

**Background and Purpose:**

Hyperoxia-induced acute lung injury (HALI) is a critical life-threatening disorder characterized by severe infiltration immune cells and death of type II alveolar epithelial cells (AECII). However, little is known about the relations between immune cells and AECII in HALI. IL-17A is a pro-inflammatory cytokine mainly secreted by Th17 cells, contributing to the pathogenesis of various inflammatory diseases. The present study investigated the role of IL-17A in cell–cell communication between immune cells and AECII in HALI, and explored the therapeutic effect of salidroside (Sal, a natural anti-inflammatory agents) on HALI.

**Methods:**

Mice with HALI were induced by exposure to hyperoxia over 90% for 12 h, 24 h, 48 h or 72 h, and the optimal timing was detected by H&E and Masson staining. Ferroptosis was confirmed by detecting the levels of MDA, Fe^2+^ and GPX4, and the morphological alterations of AECII under transmission electron microscopy. The expression of pro-inflammatory cytokine, including IL-6, TGF-β1, IL-17A and IL-17A receptor (IL-17RA) were measured by Western blotting and immunohistochemical stanning. The ferroptosis-related Act1/TRAF6/p38 MAPK pathway was detected by Western blotting. The role of pro-inflammatory cytokine IL-17A for AECII ferroptosis, and the effect of Sal on HALI were investigated by administration of Y-320 (IL-17 inhibitor) and Sal respectively 3 days before mice exposed to hyperoxia.

**Results:**

Mice exposed to hyperoxia for 24 h suffered sufficient HALI with inflammatory cell infiltration and collagen deposition, and exhibited features of ferroptosis under TME. Meanwhile, compared with sham mice, mice exposed to hyperoxia showed down-regulation of GPX4, and up-regulation of IL-6, TGF-β1, IL-17A, IL-17RA, Act1, TRAF6, p38 MAPK and p-p38 MAPK. Moreover, inhibition of IL-17A with Y-320 or administration with Sal could reverse the effect caused by hyperoxia respectively.

**Conclusions:**

IL-17A is associated with immune cells infiltration in HALI, and contributes to ferroptosis of AECII that related to Act1/TRAF6/p38 MAPK pathway. Additionally, Sal protects against HALI throughout the whole pathogenic process.

**Video Abstract**

**Supplementary Information:**

The online version contains supplementary material available at 10.1186/s12964-022-00994-1.

## Plain English summary

Oxygen inhalation has been widely used in the treatment of some diseases caused by hypoxia. This often leads people to mistakenly believe that oxygen inhalation is beneficial without harm. However, long-term high concentration oxygen inhalation will cause serious harm to the human body, sometimes even fatal. Hyperoxia causes lung cells to secrete proinflammatory factors, which promote the differentiation of infiltrated immune cells. The differentiated immune cells in turn act on lung cells and lead to their death. In short, this process is a vicious circle. Our research explores this process and is committed to finding a drug to reduce the damage of hyperoxia to the lungs when oxygen must be inhaled.

**Supplementary Information:**

The online version contains supplementary material available at 10.1186/s12964-022-00994-1.

## Background

Supra-physiological fraction of inspired oxygen is essential for hospitalized patients with hypoxemic respiratory failure. Oxygen supplementation is the most frequently administered therapy, which can be beneficial in the short-term, but not without risks in the long-term, it will be cause hyperoxia-induced acute lung injury. [1, 2] HALI is at high risk for oxidative stress as it interfaces with various airborne oxidants including air pollutants and high concentrations of oxygen (O_2_) when used as combined therapy [3]. The clinical features of HALI include the tissue infiltration of inflammatory cells, pulmonary edema, and arterial hypoxemia, which damage the vascular endothelium and alveolar epithelium, thus diminishing lung functions [4]. More and more evidence indicate that acute lung injury is closely related to nonapoptotic pathways, including pyroptosis [5, 6] and ferroptosis [7, 8]. However, whether infiltration of inflammatory cells contributing to ferroptosis in HALI is unknow, and drug exploitation for HALI is urgently needed.

Salidroside [Sal, 2-(4-Hydroxyphenyl)ethyl β-D-glucopyranoside, C14H20O7: 300.30] is the bioactive component of Rhodiola rosea. Its effects of anti-oxidation [9], anti-cancer [10] and immune regulation [11] have been widely confirmed. Numerous studies demonstrate that Sal was involved in the treatment of many diseases. More recently, it was reported that Sal can protect against acute lung injury [12], and maintain the balance between Th17 and Treg cells [9], showing potential clinical value for HALI. However, the effects and potential molecular mechanism of Sal on HALI remains unknown.

In the presence of interleukin (IL)-6 together with TGF-β make naïve CD4 + T cells differentiate into Th17 cells. As an important pro-inflammatory factor, IL-17A is mainly secreted by Th17 cells and is closely related to many diseases, including autoimmune disease [13] and acute lung injury [14]. Furthermore, IL-17A play an important role in different forms of cell death, including apoptosis [15], and pyroptosis [16]. Ferroptosis is a recently recognized form of regulated cell death (RCD) which mainly results from iron-dependent lipid peroxidation [17]. Ferroptosis has been reported to have closely associations with various lung diseases, including ischemia/reperfusion-induced acute lung injury [8], pulmonary fibrosis [18] and lung cancer [19]. However, whether IL-17A is associated with ferroptosis and together participate in HALI are unclear.

The p38 mitogen-activated protein kinase (MAPK) are activated by a variety of pro-inflammatory and environmental stressors and regulate multiple cell processes ranging from cell survival to cell death, such as inflammation and ferroptosis [20, 21]. As the key component of most signal transduction pathways and the most important common pathway of inflammation and ferroptosis, the importance of p38 MAPK is self-evident. Act1/TRAF6 is a classic signaling pathway of inflammatory cytokine IL-17A and upstream signal of p38 MAPK, plays an important regulatory role in the activation of p38 MAPK. [22]

Therefore, this study, for the first time to date, aim to demonstrate whether Sal alleviates inflammation and ferroptosis in HALI. Furthermore, we aimed to further investigate the anti-inflammatory and anti-ferroptotic effects of Sal to elucidate the role of the Act1/TRAF6/p38 MAPK signaling pathway both in the inflammation and cell death.

## Methods

### Animals

Eight-week-old KM mice (purchased from the animal center of Jinzhou University, Liaoning, China) were used to conduct in vivo experiments. Mice were housed under controlled temperature (20 °C ± 2 °C) and humidity (60% ± 10%) on a 12 h light/dark cycle. Animals were fasted for 48 h before experiments and give free access to water. All experiments were conducted in accordance with the National Institutes Health guidelines and approved by the laboratory Animal Ethics Committee of the Jinzhou Medical University for Animal Research.

### Hyperoxia-induced lung injury

In our study, the 32 mice were randomly divided for four groups (n = 8 per group): (1) room-air-expose (sham), (2) hyperoxia-expose with Sal (Sal + Hyperoxia), (3) hyperoxia-exposed (Hyperoxia), (4) hyperoxia-exposed with Y-320 (an inhibitor of IL-17) (Y-320 + Hyperoxia). The mice exposed to normoxia groups were placed in room air with 21% oxygen, and the mice exposed to hyperoxia were placed in over 90% oxygen for 24 h. The continue exposure to over 90% oxygen was achieved in a self-made airtight box which attached to a medical oxygen cylinder, and the O_2_ level inside was continuously monitored with O_2_ analyzer, mice had free access to food and water. In the first three days before exposure to the hyperoxia, mice in the Sal + Hyperoxia group or Y-320 + Hyperoxia group were treated with Sal (100 mg/Kg) or Y-320 (2 mg/Kg) once orally every day, while the rest of groups were given equal isotonic saline. Based on the above experiments, eight 8-week-old KM mice were randomly divided into two groups: Sal + Hyperoxia group and Sal + Hyperoxia + IL-17A group. Sal + Hyperoxia + IL-17A group, mice were i.v. injected with 50 μg/kg of recombinant mouse IL-17A (210–17, Pepro Tech, USA). Animal were sacrificed following reperfusion, and lungs were stored at − 80 °C until further experimental analysis.

### Lung wet/dry weight ratio

Fresh lung tissue was drained of surface moisture and weighed immediately to obtain the wet weight. Then the lungs were placed in a dry oven for 60 °C for 48 h until the dry weight was obtained. The wet to dry ratio was calculated to reflect the degree of lung edema.

### Histopathological scoring of lung injury

Lung tissues were lavaged with saline, dissected and fixed in 4% paraformaldehyde solution at 4 °C for 48 h. Embedded 5 µm tissue sections were deparaffinized with xylene followed by hydrated through graded alcohols. Then the sections were stained with hematoxylin and eosin. Histological sections were scored blindly by 5 readers to the level of induced injury. Briefly, (1) vascular congestion, (2) hemorrhage, (3) alveolar and interstitial inflammation, and (4) thickening of the alveolar wall were each scored on a scale of 0–4. 0, normal lung; 1, injury involving less than 25% of the lung; 2, injury involving 25–50% of the lung; 3, injury involving 50–75% of the lung; and 4, injury involving 75% or more of the lung. The sum of scores were calculated.

### Masson trichrome staining

Masson staining was used for detection of collagen fibers in lung tissues. The lung tissue sections were deparaffinized with xylene and followed by rehydrated in a graded alcohol. Tissues sections were stained using the Masson Trichrome Staining kit (G1340, Solarbio) following the manufacture’s instruction. After stained, the tissue sections were observed under a light microscope.

### Immunohistochemistry

Lung tissue sections were deparaffinized followed by hydrated. And then antigen was retrieved using 10 mM sodium citrate and endogenous peroxidase activity was quenched with 3% H_2_O_2_ for 10 min in the dark. Sections were washed with PBS and then blocked with sheep serum (ZSGB-BIO, ZLI-9056) for 30 min. Every slide was incubated respectively with mouse monoclonal primary antibody: IL-17RA (Bioss, bs-2606R) diluted with TBST in a ratio of 1:300 and secondary antibody: Biotin-Goat Anti Rabbit IgG (proteintech, SA00004-2) diluted with TBST in a ratio of 1:200. After washing, slides were incubated with DAB (ZSGB) followed by stained with hematoxylin and then observed under a light microscope.

### Immunofluorescence staining

Lung tissue sections were deparaffinized and rehydrated, and then heated for antigen retrieval in the citrate buffer. After that, they were permeabilized with 0.1% Triton X-100, and blocked with 5% goat serum before incubation with anti-IL-17 (WL02981, 1:200, Wanleibio) and anti-GPX4 (ab125066, 1:200, Abcam) antibodies overnight at 4 °C. Then, the sections were leaved at room temperature for 2 h. After five times washing in phosphate buffered saline (PBS), these sections were incubated with green-fluorescent Alexa 488 goat anti-mouse IgG antibody (K1204, 1:400, Apexbio) and red-fluorescent Dylight 649 goat anti-rabbit IgG antibody (E032620, 1:400, Earthox) for 2 h at room temperature. Counterstaining of nuclei with DAPI (ab228549, Abcam) was also performed. Then slides were observed, and the images were captured with a fluorescence microscope.

### Transmission electron microscopy

The lung tissues were fixed for 2 h in 2.5% glutaraldehyde in a 0.05 M sodium cacodylate buffer at a pH of 7.2 at 25 °C, followed by 2 h in 1% OsO4 in a 0.1 M sodium cacodylate buffer and 18 h in 1% aqueous uranyl acetate. After dehydration through an ethanol series, the specimens embedded in Epon 812 and ultrathin sections were collected on copper grids. After staining with uranyl acetate and lead citrate, the sections were examined using a Jeol1230 transmission electron microscope.

### Western blotting

Lung tissue samples of 4 different groups were dissected and homogenized in RIPA lysis buffer (Solarbio, R0010) containing with phenylmethanesulfonyl fluoride (PMSF; Solarbio, P0100). Place the samples on ice to sufficiently lysis it and then the homogenate was centrifuged for 30 min at 4 °C. After centrifugation, supernatants were collected and the protein concentration was measured using a BCA kit (Solarbio, PC0020). The proteins were denatured at 100 °C for 5 min. Protein samples (20 ug/lane) were separated on a 10% SDS‐polyacrylamide gel by electrophoresis and transferred to a polyvinylidene fluoride (PVDF) membrane. The PVDF membrane was blocked with 5% nonfat milk at room temperature for 2 h and diluted in TBST, and further incubated with primary antibodies overnight at 4 °C. Herein, various primary antibodies were: rabbit monoclonal anti-GPX4 (ab125066, abcam, 1:2000), anti-TRAF6 (ab40675, abcam, 1:2000), anti-IL-17A (ab79056, abcam, 1:2000), anti-IL-17RA (bs-2606R, Bioss, 1:1000), anti- Act1 (A6776, ABclonal, 1:2000), anti-IL-6 (bs-6309R, Bioss, 1:1000), anti-TGF-β1 (orb11468, Biorbyt, 1:200), anti-p38 MAPK (AF6456, Affinity, 1:1000), anti-phospho-p38 MAPK (AF4001, Affinity, 1:1000), anti-β-actin(E021020, Earthox, 1:5000). After washing 4 times with TBST for 20 min, the membranes were incubated with anti-rabbit horseradish peroxidase-conjugated secondary antibodies. Finally, the protein bands were detected using an ECL detector. Band density was quantified by Image J software.

### Iron assay

The concentrations of endogenous ferrous iron levels of the lung tissue were measured with the iron assay kit (I291, Dojindo, Japan). Lung tissue was fully washed with cold PBS, and homogenized in iron assay buffer on the ice, and then centrifuged at 16,000 × g for 10 min. The supernatant was collected for subsequent experiments. The 100 μL standard (1 mmol/L) was added with 900 μL assay buffer to made into 1000 μmol/l standard solution and then diluted to prepare standard solution (50, 25, 12.5, 6.25, 3.125, 1.5625, 0 μmol/l). The 5% volume of reducer solution was added to each of standard solution, and 5% volume of assay buffer for ferrous iron along with blank tube. All tubes were incubated for 15 min at 37 °C. The 100 μL iron probe was added to each reaction, then mixed and incubated for a further 60 min at 37 °C in a 96-well plate. The absorbance at 593 nm was measured by microplate reader to calculate the iron level.

### MDA assay

In order to evaluate the level of ferroptosis in each group, the concentration of malondialdehyde (MDA) in cell lysates was assessed with MDA assay kit (WLA048, Wanleibio, China) according to the manufacture’s instruments.

### Statistical analysis

The results are presented as mean ± SEM. Differences were analyzed using an unpaired two-sided Student’s t-test for a two-group comparison or one way ANOVA test followed by a Bonferroni post hoc test for multiple comparisons. *P* values < 0.05 were considered to indicate a statistically significant difference. Statistical analyses were carried out with SPSS 20.0.

## Results

### Hyperoxia induces acute lung injury

**Fig. 1:**
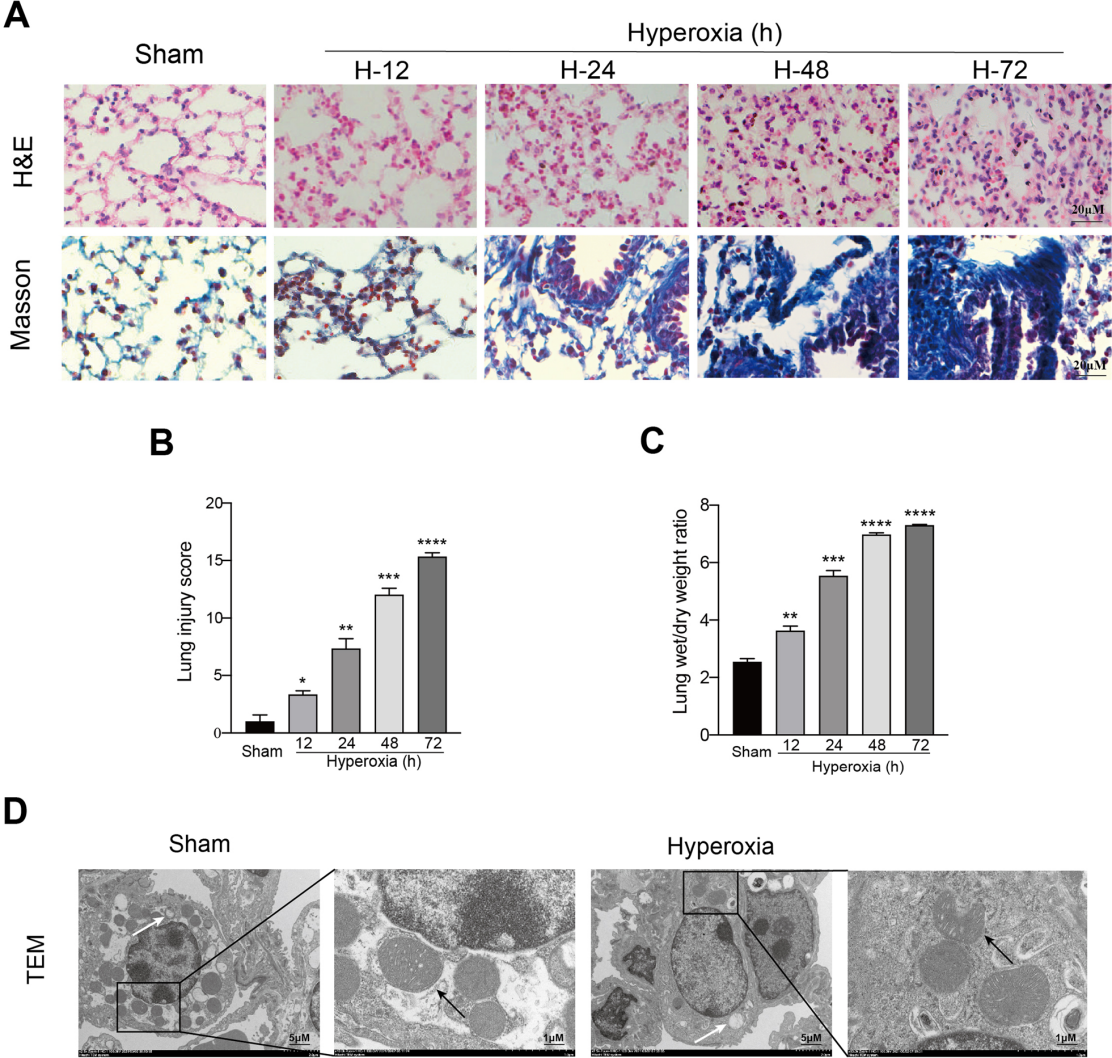
Hyperoxia induces acute lung injury. Mice were exposed to over 90% oxygen for 12, 24, 48, 72 h respectively as indicated. Sham mice were included as controls. **a** Representative H&E- and Masson trichrome-stained lung sections. Morphology was examined using light microscopy. Scar bar = 20 μM. **b** Histopathological scoring of lung injury was analyzed in each group. **c** Lung wet/dry weight ratio was analyzed at varying times exposure to over 90% oxygen. **d** TEM images of AECII both in sham and hyperoxia (24 h) groups. The white arrow indicates lamellar body. The black arrows indicate mitochondria. Images are representative of three independent experiments. Data are shown as mean ± SEM. n = 3 per group. ^*^P < 0.05, ^**^P < 0.01, ^***^P < 0.001. ^*^Compared with the sham group

To verify the existence of acute lung injury, KM mice were treated with hyperoxic oxygen (concentration is more than 90%) in different hours (sham, 12 h, 24 h, 48 h, 72 h). H&E staining of lung showed that, with increasing duration of hyperoxia treatment, edema, hemorrhage, infiltration of immune cells, and thickening of the alveolar wall were aggravated, as compared with the observations in sham mice (Fig. 1a). Correspondingly, histopathological scoring of lung injury was analyzed in each group (Fig. 1b). Also, Masson’s staining was used to assess pulmonary fibrosis. With increasing of hyperoxia duration, it’s showed more collagen deposit than those in sham mice. The results of lung wet to dry ratio showed that pulmonary edema gradually aggravated with the increase of hyperoxia exposure time (Fig. 1c). Since the histological structures of the lung suffered sufficient changes with 24 h hyperoxic oxygen treatment, the ultrastructure changes of alveolar epithelial cells were detected with transmission electron microscopy (TEM) in mice with 24 h hyperoxic oxygen treatment. We found that compared with the results in sham mice, the mitochondrial morphology in AECII (characteristic organelle: lamellar body) of mice in the hyperoxia group (24 h) underwent features of ferroptosis, including the presence of small mitochondria with condensed mitochondrial membrane densities, reduction or vanishing of mitochondria cristae, as well as outer mitochondrial membrane rupture (Fig. 1d). In conclusion, inspire of high concentration of oxygen induces acute lung injury.

### Hyperoxia enhances inflammation in acute lung injury

**Fig. 2:**
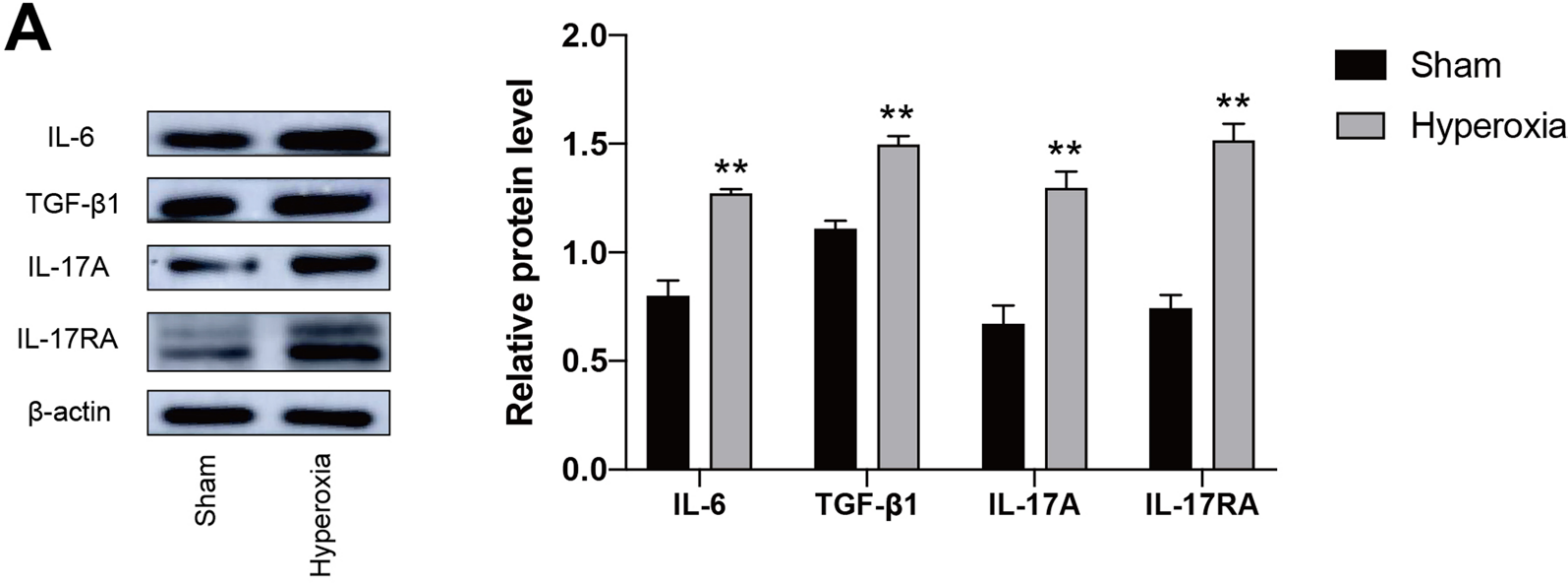
Hyperoxia enhances inflammation in acute lung injury. Mice were randomly divided into two groups: sham and hyperoxia. Mice in hyperoxia group were exposed to over 90% oxygen for 24 h. **a** IL-6, TGF-β1, IL-17A and IL-17RA protein levels in lung tissues were measured by western blotting. Histogram shows densitometry quantification of IL-6, TGF-β1, IL-17A and IL-17RA normalized to β*-*actin. Data are shown as mean ± SEM. n = 3 per group. ^*^P < 0.05, ^**^P < 0.01, ^***^P < 0.001. ^*^Compared with the sham group

Acute lung injury caused by infection and ischemia/reperfusion is often accompanied by severe lung inflammation [4, 23]. Since the immune cells infiltrated significantly in HALI, the alterations of inflammatory cytokines were tested by western blotting. The protein levels of IL-6 and TGF-β1 in hyperoxia group (exposure to over 90% oxygen for 24 h) significantly increased, compared with the results in sham mice. As the above described, IL-6 combined with TGF-β together promoted the production of IL-17A [13, 14]. And the protein levels of IL-17A and its receptor IL-17RA in hyperoxia group also significantly increased, compared with the results in sham mice (Fig. 2a). These results indicated that the production of pro-inflammatory cytokine IL-17A may be ascribed to the accumulation of IL-6 and TGF-β1 in HALI.

### Inflammation in HALI is associated with ferroptosis

**Fig. 3:**
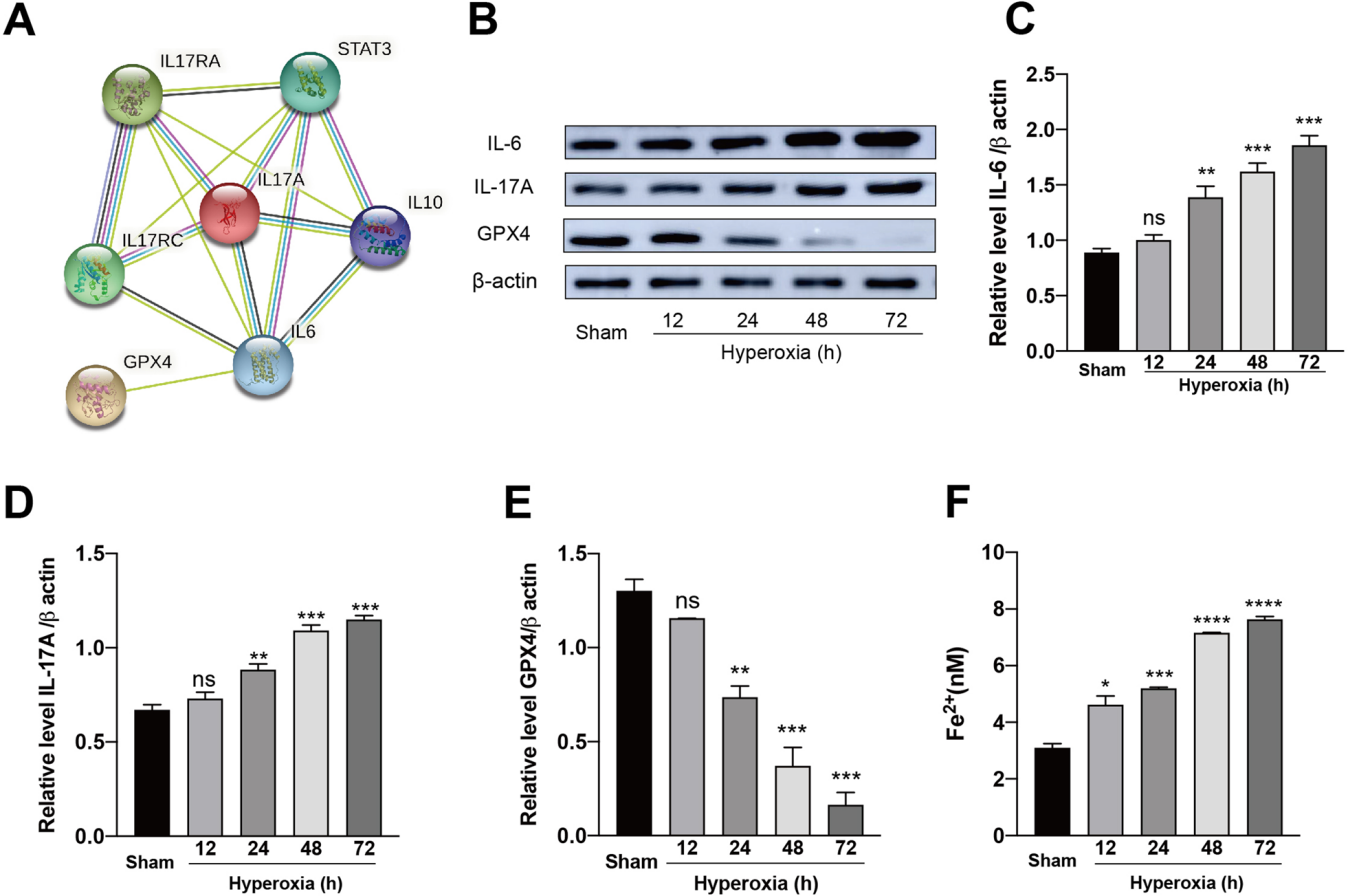
Inflammation in HALI is associated with ferroptosis. **a** PPI network of IL-17A and GPX4. **b**–**e** Mice were exposed to over 90% oxygen for 12, 24, 48, 72 h respectively. Sham mice were included as controls. The protein expression levels of IL-6, IL-17A and GPX4 of lung tissues were analyzed by western blotting. **f** Endogenous levels of Fe^2+^ of the lung tissue in different groups were measured with an iron assay. Data are shown as mean ± SEM. n = 3 per group. ^*^P < 0.05, ^**^P < 0.01, ^***^P < 0.001. ^*^Compared with the sham group

Ferroptosis is a newly discovered RCD, which is characterized by iron overload and GPX4 depletion. To investigate the relations between inflammation and ferroptosis, IL-17A and GPX4 were uploaded to String 11.0 database (https://stri-ng-db.org) [24], and the species was set as “Homosapiens” to obtain the protein–protein interactions (PPI). The result showed that IL-6 is the main protein connecting IL-17A and GPx4 (Fig. 3a). Next, we used western blotting to detect the expression of IL-6, IL-17A and GPX4. The levels of IL-6 and IL-17A were significantly increased with increasing hyperoxia duration, as compared with the results in the sham mice (Fig. 3b–d). Meanwhile, the expression of GPX4 was significant decreased with increasing hyperoxia duration, as compared with the result in sham group (Fig. 3b and e). The endogenous ferrous iron level was tested in each group. As the duration of hyperoxia increased, we found that Fe^2+^ was significantly increased compared with the sham group (Fig. 3f). Overall, our results indicated that inflammatory cytokines such as IL-17A is closely associated with ferroptosis in HALI.

### *Inhibition of ferroptosis *via* IL-17A contributes to Sal-alleviated HALI*

**Fig. 4:**
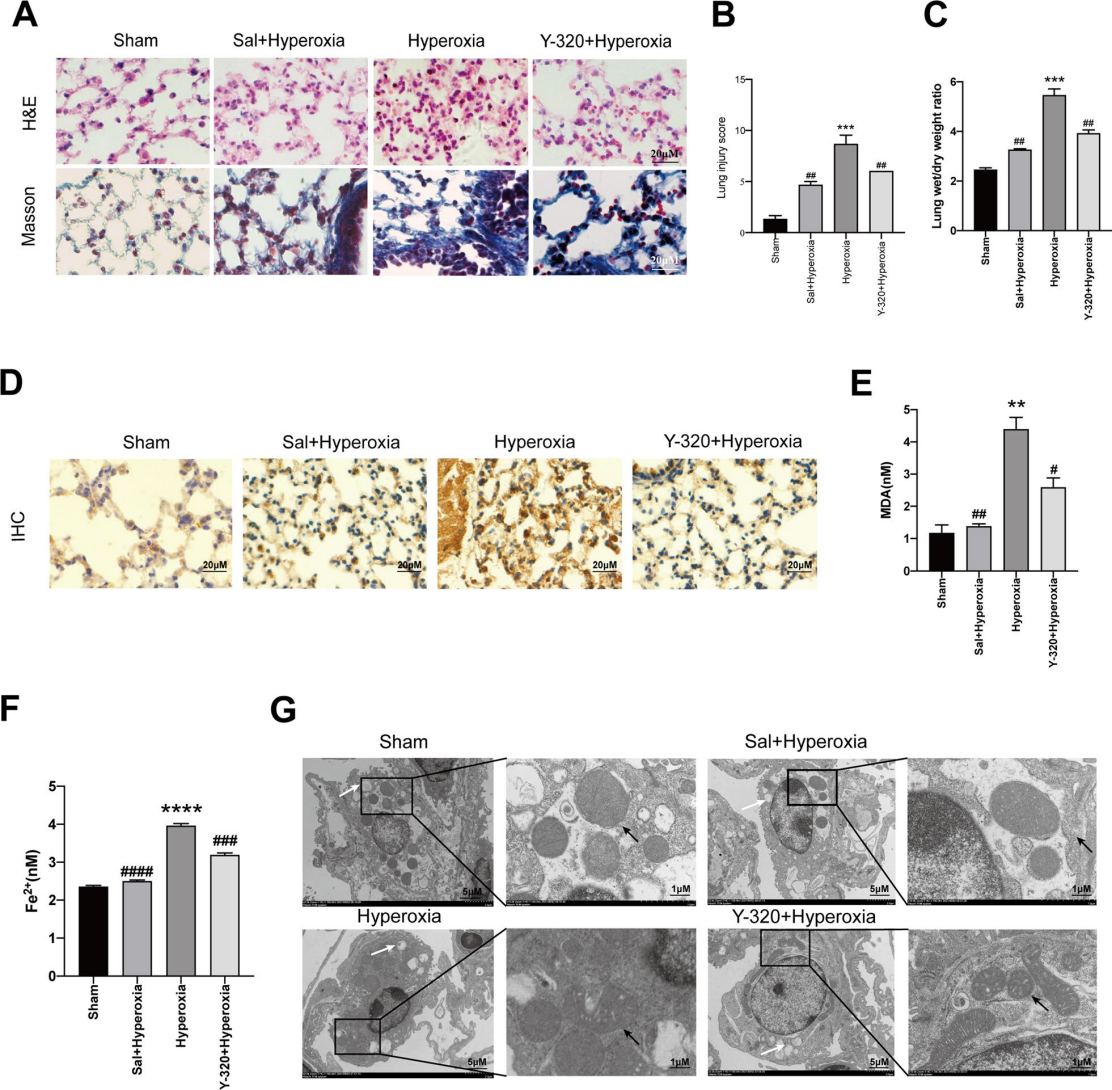
Inhibition of ferroptosis via IL-17A contributes to Sal-alleviated HALI. KM mice were divided into four groups: sham, Sal + hyperoxia, hyperoxia, Y-320 + hyperoxia. Mice were exposed to over 90% oxygen for 24 h, in the first three days before exposure to the hyperoxia, the Sal group mice were treated with Sal (100 mg/Kg), and the Y-320 group mice were treated with Y-320 (2 mg/Kg) once orally every day, while the rest of groups were given equal isotonic saline. **a** Representative H&E- and Masson trichrome-stained lung sections after different treatments. Morphology was examined using light microscopy. Scar bar = 20 μM. **b** Lung injury score was tested in each group. **c** The lung wet/dry weight ratio was analyzed in each group as indicated. **d** The expression and location of IL-17RA were detected by immunohistochemistry in each group. Morphology was examined using light microscopy. Scar bar = 20 μM. **e** The MDA level in each group was measured by MDA assay. **f** Endogenous levels of Fe^2+^ of the lung tissue in various groups were measured with an iron assay. **g** Transmission electron microscopy images of lung tissues in each group. The white arrow indicates lamellar body. The black arrows indicate mitochondria. Scale bars: 5 μM; 1 μM. Data are shown as mean ± SEM. n = 3 per group. ^*^P < 0.05, ^**^P < 0.01, ^***^P < 0.001, ^**#**^*P* < 0.05, ^**##**^*P* < 0.01, ^**###**^*P* < 0,001 between the groups. ^*^Compared with the sham group. ^**#**^Compared with the hyperoxia group

The morphological changes and wet/dry weight ratio of lung were studied to explore the effects of Sal on HALI. Furthermore, IL-17A was inhibited by Y-320 to reveal the underlying mechanisms. Iron deposition in alveolar epithelial cells leads to severe cell injuries which ultimately promote the development of pulmonary fibrosis [18]. Mice in hyperoxia group showed edema, atelectasis, necrosis, alveolar and interstitial inflammation, increased collagen deposits, and elevated wet to dry ratio of lung tissue, while these pathological changes were reversed by Sal or Y-320 respectively in Sal + Hyperoxia group and Y-320 + Hyperoxia group (Fig. 4a–c). Immunohistochemistry for IL-17RA showed that there is a significantly increased in hyperoxia group compared with the mice in sham group, but the effect was reversed by Sal or Y-320 treatments respectively (Fig. 4d). The levels of MDA and Fe^2+^ are closely related with ferroptosis. Therefore, levels of MDA and Fe^2+^ were investigated in addition to the ultrastructure of alveolar epithelial cells. In the present study, the levels of MDA and Fe^2+^ were significantly increased in the hyperoxia model compared with sham mice. Additionally, the mitochondrial morphology in AECII of mice in the hyperoxia group underwent the characteristics of ferroptosis. Interestingly, the alterations of MDA, Fe^2+^ and ultrastructure of alveolar epithelial cells were reversed either by inhibition of IL-17A with Y-320 or by Sal respectively (Fig. 4e–g).

### Act1/TRAF6/p38 MAPK pathway contributes to IL-17 mediated ferroptosis in HALI

**Fig. 5:**
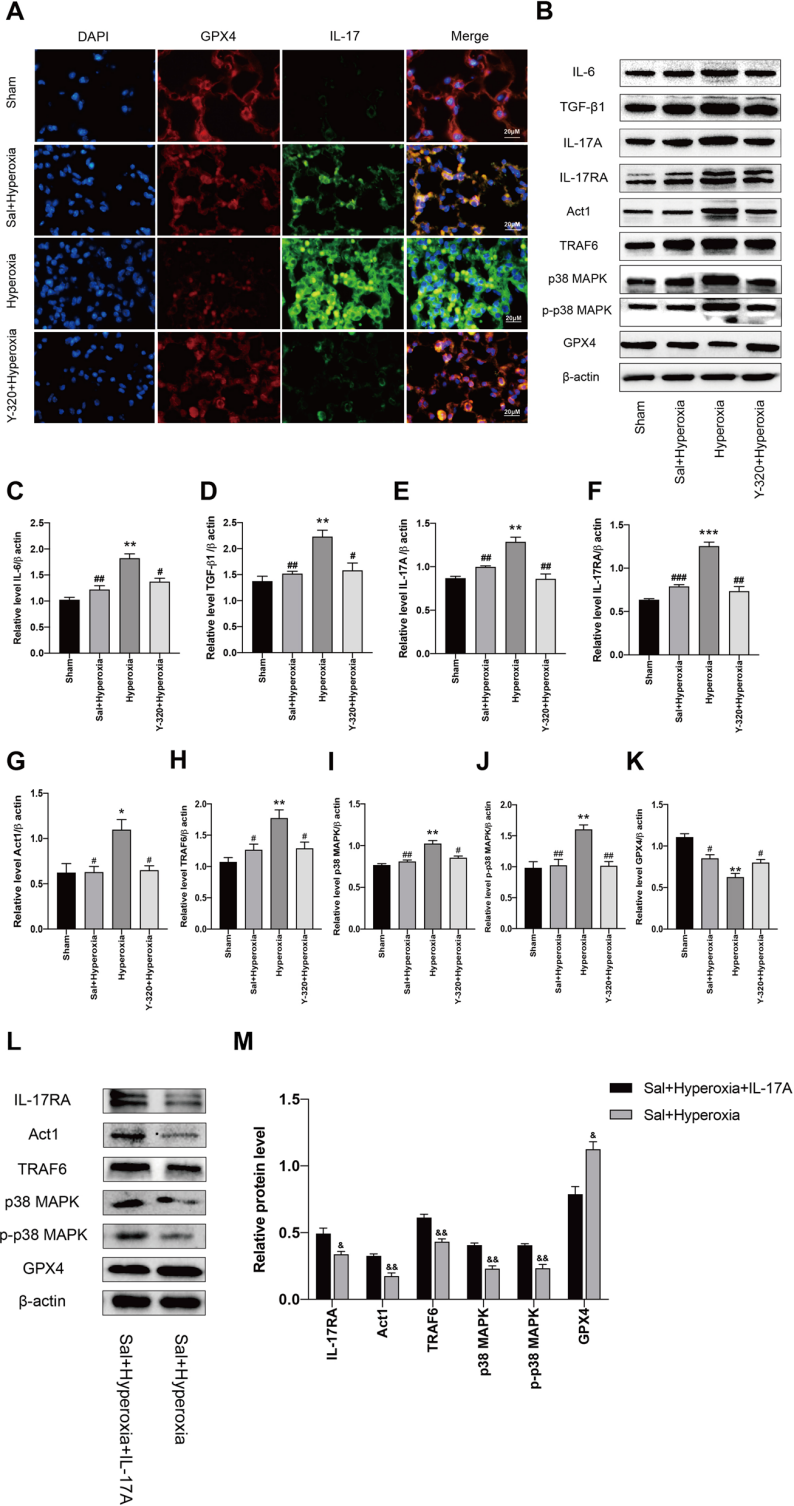
Act1/TRAF6/p38 MAPK pathway contributes to IL-17 mediated ferroptosis in HALI. **a** Immunofluorescence staining of lung tissues to detect the expression and location of GPX4 (red) and IL-17 (green) in each group. Scar bar = 20 μM. Nuclei were stained with DAPI (blue). **b**–**k** IL-6, TGF-β1, IL-17A, IL-17RA, Act1, TRAF6, p38 MAPK, p-p38 MAPK, GPX4 protein levels in each group were demonstrated by western blotting. **l**–**m** IL-17RA, Act1, TRAF6, p38 MAPK, p-p38 MAPK, GPX4 protein levels in Sal + Hyperoxia and Sal + Hyperoxia + IL-17A groups were demonstrated by western blotting. Data are shown as mean ± SEM. n = 3 per group. ^*^P < 0.05, ^**^P < 0.01, ^***^P < 0.001, ^**#**^*P* < 0.05, ^**##**^*P* < 0.01, ^**###**^*P* < 0,001, ^&^*P* < 0.05, ^&&^*P* < 0.01 between the groups. ^*^Compared with the sham group. ^**#**^Compared with the hyperoxia group. ^&^Compared with the Sal + Hyperoxia group

As we mentioned above, the anti-ferroptotic effect of Sal is closely related to IL-17A. And the immunofluorescence staining showed that Sal or Y-320 could significantly reduce the expression of IL-17 and significantly increase the expression of GPX4 in the hyperoxia group compared with sham group (Fig. 5a).

The p38 MAPK is typically upregulated in cancer, inflammation and ferroptosis [20, 21]. Therefore, the relationship between p38 MAPK and Sal-reversed ferroptosis is investigated. The results showed that the increase of IL-17A, IL-6 and TGFβ1, and decrease of GPX4 were accompanied with increase of p38 MAPK, (p)-p38 MAPK and Act1/TRAF6 (an upstream of p38 MAPK) in the hyperoxia group, compared with sham group (Fig. 5b–k). While these alterations of hyperoxia-treated mice were reversed by Y-320 or Sal respectively. The above results suggest that Act1/TRAF6/p38 MAPK signaling pathway was involved in IL-17A-mediated ferroptosis, which participates in the protective effects of Sal on HALI.

To further verify the role of Act1/TRAF6/p38 MAPK in ferroptosis of HALI, we applied recombinant IL-17A in hyperoxia-expose with Sal administration (Sal + Hyperoxia) group mice and compared it with Sal + Hyperoxia group mice. We found the protein levels of IL-17RA, Act1, TRAF6, p38 MAPK, p-p38 MAPK in Sal + Hyperoxia + IL-17A group significantly increased compared with the results in Sal + Hyperoxia group. Meanwhile, compared with the result in Sal + Hyperoxia group, the level of GPX4 was significantly decreased in Sal + Hyperoxia + IL-17A group (Fig. 5l–m).

## Discussions

Sal, a phenylpropanoid glycoside compound, is an active ingredient form Rhodiola rosea, named for its red color in the soaking solution of its flowers, roots and stems. Its effect of anti-fatigue, anti-cancer, anti-apoptosis and anti-inflammation have been widely confirmed [9, 10, 12], but the effects and mechanism on HALI is still unclear. In this study, a hyperoxic model of mouse was successfully established by inspire over 90% oxygen, and the anti-ferroptotic effect of Sal on HALI was investigated. We postulated that increased IL-6 and TGF-β1 by AECII promotes the release of IL-17A from infiltrated immune cell, and then in turn induce the ferroptosis of AECII in HALI via Act1-TRAF6-MAPK pathway, which contributes to the protective effects of Sal on HALI. This is the first to investigate the cell–cell interaction between immune cells and AECII mediated by IL-17A in HALI, which provides a novel insight for the clinical treatment of Sal in HALI.

Acute lung injury is a critical life-threatening disorder characterized by acute severe hypoxia that is caused by onset of pulmonary inflammation due to infection, surgery, burns and ischemia/reperfusion [4, 25]. Commonly, supraphysiological oxygen concentration is needed to ensure adequate blood oxygenation. However, oxygen supplementation of high concentrations is a double-edged sword. On the one hand, O_2_ plays an important role in lifesaving; On the other hand, continuous expose to hyperoxia causes disturbances in the pulmonary system and gas exchange impairment. In the present study, pathological alterations of HALI were confirmed by H&E and Masson trichrome staining after 24 h over 90% oxygen supplementation. What’s more, there are typical mitochondria changes of ferroptosis occurred in AECII of HALI model, including the presence of mitochondria smaller than normal with increased membrane density, reduction or vanishing of mitochondria crista, as well as outer membrane rupture which are consistent with the results reported by S. J. Dixon [17]. Researchers have demonstrated that continuously exposure in hyperoxia environment exaggerated pulmonary inflammation, and promoted the secretion of cytokines by AECII, such as IL-6 and TGF-β that play a very important role in acute lung injury [2, 7, 26]. AECII are an important part of airway epithelial cells which product and recycle surfactant to balance alveolar surface tension, and hyperplasia in reaction to acute lung injury, serving as the progenitor for type I alveolar epithelial cells [27, 28]. Coincide with these studies, we found that HALI exhibits ferroptosis together with the accumulation of IL-6 and TGF-β1.

As we known, IL-6 together with TGF-β released from AECII make the naïve CD4 + T cells differentiate into Th17 cells, and effect the balance of Th17/Treg cell [13, 22]. Th17 cells secretes various inflammatory cytokines, among which, IL-17A plays an important role in oxidative inflammation by binding to its receptors on AECII [29]. The IL-17A/IL-17RA axis has been reported to participate in several acute and chronic lung diseases [22, 30]. In our study, we demonstrated that the expression of IL-17A significantly increased, and the level of IL-17RA on AECII increased correspondingly in the hyperoxia group. Interestingly, expression of IL-17RA correlated to IL-17 was also reported by other studies [31, 32]. However, the role of IL-17A/IL-17RA axis for ferroptosis remains uncertain. Ferroptosis is recently discovered iron-dependent form of RCD which is characterized by accumulation of lipid products MDA and Fe^2+^, and decrease of GPX4 [17, 33, 34]. In the present study, mice in the hyperoxia group showed ferroptotic changes of mitochondria, elevation of Fe^2+^ and MDA were increased, while decreased GPX4. We found that inhibition of IL-17A with Y-320 reversed the ferroptosis and attenuated the pathological alterations of HALI. Clinically, the therapeutic and deleterious effects of high concentrations of inspired oxygen are difficult to managed. There is a need to improve prevention and therapies to alleviate HALI. In our study, we found that administration of Sal exhibited similar effects to Y-320 on HALI. Therefore, we deduced that IL-17A contributes to ferroptosis in HALI, which participates in Sal alleviating HALI. Overall, these findings suggest that IL-17A plays an important role for the cell–cell interaction between filtrated immune cells and AECII.

p38 MAPK is an important member of the MAPK family, and its pathways plays an important role both in regulating the activity and expression of key inflammatory mediators and ferroptosis [20, 21, 35]. The Act1/TRAF6 signaling pathway is an important part of IL-17 signaling pathway. Act1 is cytoplasmic protein that shares homology to the cytoplasmic domain of IL-17R family, and it have a direct interaction with IL-17RA [36]. TRAF6 is an adapter protein that associates indirectly with IL-17RA through Act1, and recruitment of Act1 and TRAF6 to IL-17RA further activates p38 MAPK. [22] It has been reported that IL-17A mediates p38 MAPK activation via Act1/TRAF6 signaling in cardiomyocytes [37]. Therefore, in this study, p38 MAPK was proposed to be the tie linking IL-17A signaling to ferroptosis. We further investigated the potential effect of Act1/TRAF6/p38 MAPK signaling pathway on ferroptosis in HALI. In HALI mice, the Act1/TRAF6/p38 MAPK pathway was activated, as evidenced by increase of the expression of Act1, TRAF6, p38 MAPK and p-p38 MAPK (activated p38 MAPK). While, the activation of Act1/TRAF6/p38 MAPK pathway was reversed by inhibition of IL-17A or administration of Sal respectively. When we applied recombinant IL-17A in Sal + hyperoxia group mice, the protein levels of IL-17RA, Act1, TRAF6, p38 MAPK and p-p38 MAPK increased significantly, and the expression level of GPX4 significantly decreased. Therefore, we demonstrated that IL-17A/IL-17RA mediates ferroptosis of AECII, least in part, via Act1/TRAF6/p38 MAPK pathway, which is responsible for the protective effects of Sal on HALI.

**Fig. 6:**
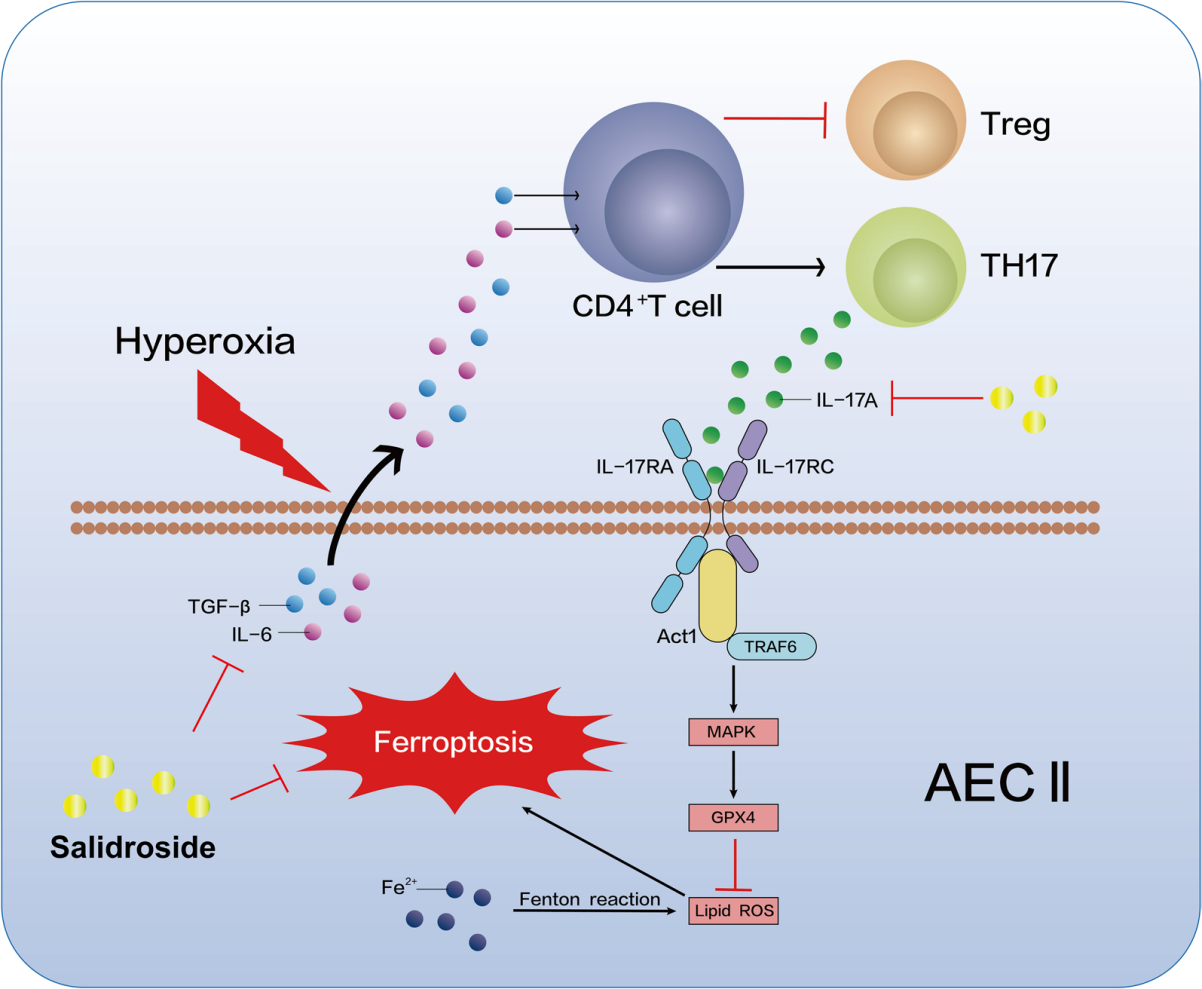
Sal attenuates hyperoxia-induced lung injury via ferroptosis by regulating Act1-TRAF6-MAPK pathway

Prevention and treatment of HALI is of great clinical importance. Both inflammation and ferroptosis contribute to HALI, our study is the first to demonstrate a link between the inflammatory factor IL-17A and ferroptosis which is a novel form of regulated cell death. Furthermore, Sal was first applied to HALI to investigate its anti-inflammatory and anti-ferroptotic effects throughout the whole pathogenic process (Graphic summery, Fig. 6). The results in this study may have a significant effect in improving the level of therapies to alleviate acute lung injury in patients undergoing oxygen therapy, by suppressing alveolar epithelial cells death. There is no corresponding experiment performed in vitro in this study. In the future, further investigating mechanism by cells will perform to reveal the potential clinical value of Sal.

## Conclusions

Taken together, we postulated that continuous hyperoxia exposure stimulates AECII to secrete IL-6 and TGF-β1, the latter two cytokines together drive the infiltrated immune cells to secrete IL-17A, which in turn induce the ferroptosis of AECII via Act1/TRAF6/p38 MAPK signaling pathway. Importantly, Sal protects against HALI throughout the whole pathogenic process.

## Data Availability

All data generated or analysed during this study are included in this published article [and its supplementary information files].

## Abbreviations

HALI: Hyperoxia-induced acute lung injury
AECII: Type II alveolar epithelial cells
Sal: Salidroside
TEM: Transmission electron microscopy
IL-17RA: IL-17A receptor
O_2_: Oxygen
RCD: Regulated cell death
MAPK: The mitogen-activated protein kinase
MDA: Malondialdehyde
